# The Relationship between the M1/M2 Macrophage Polarization and the Degree of Ossicular Erosion in Human Acquired Cholesteatoma: An Immunohistochemical Study

**DOI:** 10.3390/jcm11164826

**Published:** 2022-08-18

**Authors:** Mohamed Bassiouni, Philipp Arens, Samira Ira Zabaneh, Heidi Olze, David Horst, Florian Roßner

**Affiliations:** 1 Department of Otorhinolaryngology, Charité—Universitätsmedizin Berlin, Corporate Member of Freie Universität Berlin, Humboldt-Universität zu Berlin, and Berlin Institute of Health, 10117 Berlin, Germany; 2 Institute of Pathology, Charité—Universitätsmedizin Berlin, Corporate Member of Freie Universität Berlin, Humboldt-Universität zu Berlin, and Berlin Institute of Health, 10117 Berlin, Germany

**Keywords:** macrophages, polarization, inflammation, cholesteatoma

## Abstract

The differential involvement of the macrophage activation phenotypes (M1 vs. M2) has been linked to disease severity in various chronic inflammatory disorders. Pharmacologic manipulation of the M1/M2 macrophage polarization has shown therapeutic potential. Cholesteatoma is a destructive chronic middle ear disease with potentially life-threatening complications. The distribution of macrophage polarization phenotypes in middle ear cholesteatoma has not been described. In the present study, human cholesteatoma specimens acquired during tympanomastoidectomy were retrospectively retrieved and immunohistochemically characterized using a combination of antibodies labeling M1 macrophages (CD80), M2 macrophages (CD163), and total macrophages (CD68). The correlations between the immunohistochemical findings and clinical presentation were assessed. The findings revealed that cholesteatomas with more extensive ossicular erosion demonstrated a significantly higher number of M1 (CD80+) cells and a higher M1/M2 ratio than less invasive cholesteatomas (Wilcoxon test, *p* < 0.05). The extent of ossicular erosion correlated significantly with the M1/M2 ratio (Spearman correlation coefficient ρ = 0.4, *p* < 0.05). Thus, the degree of ossicular erosion in human acquired cholesteatoma appears to be related to the M1/M2 macrophage polarization. The investigation of macrophage polarization and functions in various clinical presentations of middle ear cholesteatoma is of great interest since it may contribute to the development of pharmaceutical treatment approaches.

## 1. Introduction

Acquired cholesteatoma is an invasive chronic disease of the middle ear with considerable morbidity and potentially life-threatening complications [1,2,3]. The pathogenesis of cholesteatoma is characterized by bacterial superinfection and bony destruction of the middle ear, inner ear, and skull base [2,4,5,6], which can lead to facial paralysis and intracranial complications [2,7]. Importantly, cholesteatoma is also a common cause of hearing loss in both children and adults [8,9,10]. The only currently available treatment for cholesteatoma is surgical removal [11,12,13]. However, acquired cholesteatoma still has high residual and recurrence rates after treatment [14] and often requires multiple ear surgeries [15]. Although several theories about the etiology of cholesteatoma have been proposed, the exact mechanism remains elusive.

**Figure 1:**
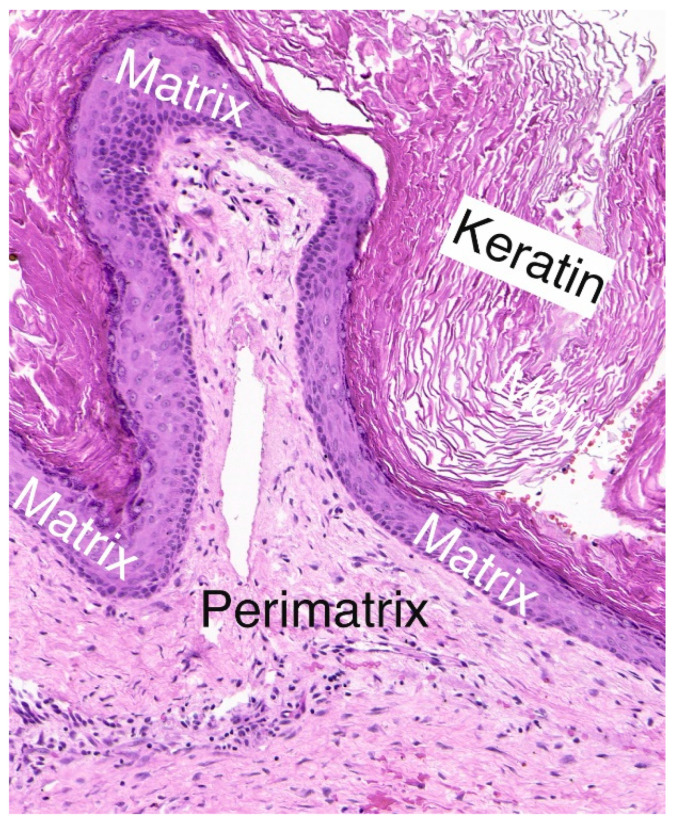
Paraffin section of a cholesteatoma specimen stained with hematoxylin and eosin (H&E) showing the two histological layers of cholesteatoma: the squamous epithelial layer (matrix) and the underlying stromal lamina propria (perimatrix). The cyst contains keratin lamellae.

Histologically, cholesteatomas consist of two main layers: (1) the matrix consists of keratinized stratified squamous epithelium similar to skin with a high proliferation index; and (2) the perimatrix consists of subepithelial stromal connective tissue with inflammatory cells (monocytes, macrophages and infiltrating leukocytes) that secrete a myriad of cytokines [4,16,17,18,19] (Figure 1). The presence of macrophages and other immune cells in cholesteatoma has been previously described [5,20,21,22]. Macrophages are a heterogeneous population of immune cells that are activated in response to injurious extracellular stimuli, most notably bacterial infection [23,24,25]. Activation of macrophages leads to their polarization into the classically activated (M1) or alternatively activated (M2) phenotype [23,24,25]. The proinflammatory M1 phenotype is characterized by the secretion of inflammatory mediators and cytokines that lead to tissue remodeling and bone destruction, while the anti-inflammatory M2 phenotype is involved in cellular repair, proliferation, and wound healing [23,24,25,26,27]. The differential involvement of the M1/M2 macrophage polarization phenotypes in chronic inflammatory diseases has been demonstrated previously [26,27]. For example, Crohn’s disease is an autoimmune inflammatory disease associated with a pathologic macrophage response that is treated by biologics such as tumor necrosis factor alpha (TNFα) inhibitors [28,29,30]. The therapeutic effect of TNFα inhibitors in Crohn’s disease may be attributed to a polarization shift of macrophages from the M1 to the M2 phenotype [28]. Thus, the investigation of the macrophage polarization profile of middle ear cholesteatoma may reveal findings of therapeutic significance. However, the M1/M2 macrophage polarization profiles in middle ear cholesteatoma and their association with the clinical presentation remain unclear. In the present study, we performed an immunohistochemical analysis of the macrophage polarization markers in cholesteatoma specimens obtained during middle ear surgery.

## 2. Materials and Methods

The study was approved by the local ethics committee of Charité Medical University (approval number EA1/182/21). The study design included the retrospective analysis of patient records, audiograms, and computed tomography (CT) image sets of juvenile and adult patients who underwent tympanomastoidectomy at the Department of Otorhinolaryngology of the Charité Campus Mitte for acquired cholesteatoma with intraoperative harvesting of a tissue biopsy. As a departmental policy, the intraoperative visual confirmation of typical cholesteatoma morphology is considered sufficient for the confirmation of the diagnosis in our center. Thus, histopathological analysis is not routinely employed but rather based on surgeon preference in selected cases. We excluded specimens that did not show representative cholesteatoma histomorphology and those with too scarce amounts of tissue. Further exclusion criteria included recurrent cholesteatoma, congenital cholesteatoma, and school-age children (up to 12 years old) since pediatric cholesteatomas were proven to show more aggressive clinical pictures [31], with potential inherent biological differences to adult cholesteatomas [32]. The paraffin-embedded tissue blocks were retrieved for retrospective immunohistochemical analysis using a panmacrophage marker (CD68), an M1 marker (CD80), and an M2 marker (CD163). The combination of antibodies against CD80 and CD163 as M1 and M2 markers, respectively, has been previously utilized in multiple immunohistochemistry studies [33,34,35]. For quantification, at least four high-power fields (HPFs) were analyzed by a certified pathologist and manually counted in duplicates. The individual HPF counts were then averaged for every marker and specimen. The assessment of temporal bone and ossicular erosion was based on the operative notes and the preoperative CT scans. For the quantification of ossicular erosion, the operated ears were classified according to a modification of the STAMCO classification [36]: o0 (no ossicles eroded), o1 (one ossicle eroded), o2 (two ossicles eroded) or o3 (three ossicles eroded). For audiometric profiling of the patients, the preoperative pure tone audiogram was used. The preoperative air conduction, bone conduction, and air-bone gap were compared at 0.5, 1, 2, and 4 kHz. Statistical analysis was performed using JMP^®^ 15 software (SAS Institute, Cary, NC, USA). Group comparisons were conducted using nonparametric testing (Wilcoxon test). Correlation analysis was performed using the Spearman (ρ) rank correlation coefficient test. A *p*-value < 0.05 was considered statistically significant.

## 3. Results

### 3.1. Patient Characteristics and Clinical Profile

A total of 28 patients met the inclusion criteria (21 males and 7 females). The average patient age was 41 ± 17 years. According to their ossicular status, patients were classified into o0 to o3 groups as described in the methods section. In this cohort, 4 patients were included in the o0 group, 4 patients were included in the o1 group, 10 patients were included in the o2 group, and 10 patients were included in the o3 group. For group analysis, the o0 and o1 groups were combined into one group of “mild ossicular erosion”, whereas the o2 and o3 groups were combined into one group of “advanced ossicular erosion”. The extent of ossicular erosion was not significantly related to the patients’ age or sex. In this cohort, 10 out of 28 patients (35.7%) showed signs of temporal bone erosion (involving the tegmen, otic capsule, or facial canal). All 10 cases with temporal bone erosion were associated with advanced ossicular erosion (o2 or o3). Contingency analysis indicated a statistically significant association between temporal bone erosion and advanced ossicular erosion (Fisher’s exact test, *p* < 0.05). Thus, further analysis focused on ossicular erosion since it occurred more frequently and is more amenable to quantitative analysis. With regard to the pure tone audiograms, the average four-frequency thresholds at 0.5, 1, 2, and 4 kHz were 47.8 (±18.1) decibels for air conduction and 21.4 (±16.9) decibels for bone conduction, resulting in an average air-bone gap of 26.3 (±11.3) decibels. Since cholesteatoma was previously shown to cause sensorineural hearing loss [37,38], the audiometric assessments mainly aimed to investigate the hypothesis of whether the more aggressive cholesteatomas are associated with worse bone conduction thresholds. There was no statistically significant difference in the average air conduction thresholds, bone conduction thresholds, or the air-bone gap between the cholesteatoma groups with mild or advanced ossicular erosion (Wilcoxon test, *p* > 0.05) (Table 1).

### 3.2. The Expression of Macrophage Markers in the Cholesteatoma Specimens

**Figure 2:**
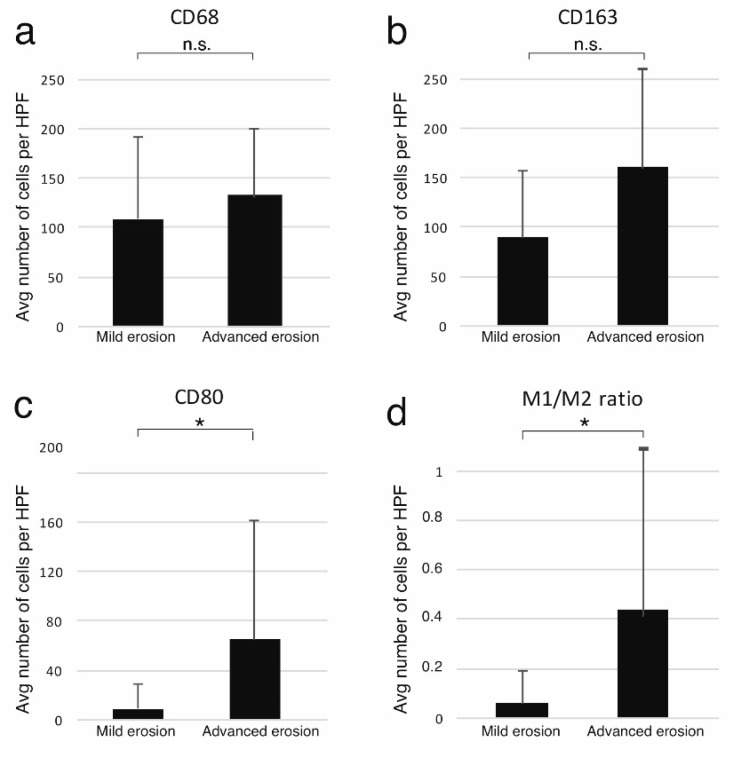
Bar graph illustration of the average number of marker-positive cells per high-power field (HPF). The bars represent the average of 28 specimens, and the error bars represent one standard deviation. n.s.: not significant. * *p* < 0.05. (**a**) Graphic illustration of the average number of CD68-positive cells per HPF in the cholesteatomas with mild (n = 8) and advanced ossicular erosion (n = 20). The difference was not statistically significant (*p* > 0.05, Wilcoxon test). (**b**) Graphic illustration of the average number of CD163-positive cells (M2 macrophages) per HPF in the cholesteatomas with mild (n = 8) and advanced ossicular erosion (n = 20). The difference was not statistically significant (*p* > 0.05, Wilcoxon test). (**c**) Graphic illustration of the average number of CD80-positive cells (M1 macrophages) per HPF in the cholesteatomas with mild (n = 8) and advanced ossicular erosion (n = 20). The difference was statistically significant (*p* < 0.05, Wilcoxon test). (**d**) Graphic illustration of the average relative ratio of CD80+ to CD163+ cells (M1/M2 ratio) in the cholesteatomas with mild (n = 8) and advanced ossicular erosion (n = 20). The difference was statistically significant (*p* < 0.05, Wilcoxon test).

**Figure 3:**
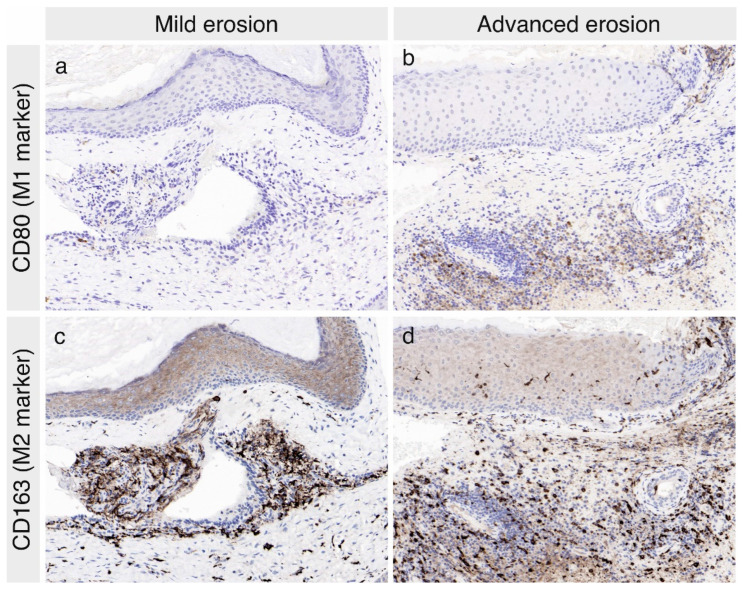
Paraffin section of a representative cholesteatoma specimen with mild erosion (o0 specimen with an intact ossicular chain) and a specimen with advanced erosion (o3) stained for the M1-marker CD80 and the M2-marker CD163. The number of CD80+ cells (M1 macrophages) was higher in the specimen with advanced erosion (**b**) compared to that with mild erosion (**a**). The number of CD163+ M2 cells was much higher than that of M1 cells in both specimens ((**a**,**b**) compared to (**c**,**d**)) without significant differences between the specimens with mild (**c**) or advanced erosion (**d**).

Next, we aimed to analyze the expression of macrophage markers in the cholesteatoma specimens. CD68 was utilized as a panmacrophage marker, whereas CD80 and CD163 were used as M1 and M2 markers, respectively. The ratio of CD80-positive to CD163-positive cells was utilized as the M1/M2 ratio, which is a commonly employed indicator of the macrophage polarization profile. The average number of positive cells per high power field (HPF) in the perimatrix for each cholesteatoma specimen was grouped according to the respective ossicular status (Figure 2). In total, M2 macrophages (CD163+ cells per HPF) were much more abundant than M1 macrophages (average CD80+ cells per HPF) (Figure 2, Figure 3). In the group analysis, the cholesteatomas with advanced ossicular erosion contained a higher number of cells expressing the M1 marker CD80 as well as a higher M1/M2 ratio than the less erosive cholesteatomas (Wilcoxon test, *p* < 0.05) (Figure 2 and Figure 3). The numbers of M2 macrophages (CD163) and total macrophages (CD68) were also higher in the cholesteatomas with advanced ossicular erosion, but the difference did not reach statistical significance (Figure 2). The correlation analysis revealed a statistically significant correlation between the extent of ossicular erosion and the M1/M2 ratio (Spearman correlation coefficient ρ = 0.4, *p* < 0.05). These findings suggest that the extent of ossicular erosion is related to the M1/M2 polarization of macrophages in acquired middle ear cholesteatoma.

## 4. Discussion

The pathophysiological hallmarks of human acquired cholesteatoma are inflammation, cell proliferation, and bone erosion [2,4,5,6]. Bacterial infections are very common in middle ear cholesteatoma since the entrapped keratin is a very suitable environment for bacterial biofilms [39,40], most commonly those containing *Pseudomonas aeruginosa* [41,42]. The tumor-like growth of cholesteatoma is attributed to unchecked cell proliferation, previously described as an aberrant wound healing process [43,44,45]. The co-occurrence of inflammatory and proliferative aspects thus appears to be characteristic of cholesteatoma [43,44,45,46] and may be attributed to macrophage plasticity since macrophages may shift between the proinflammatory M1 phenotype and the homeostatic M2 phenotype based on changes in the cholesteatoma microenvironment.

**Figure 4:**
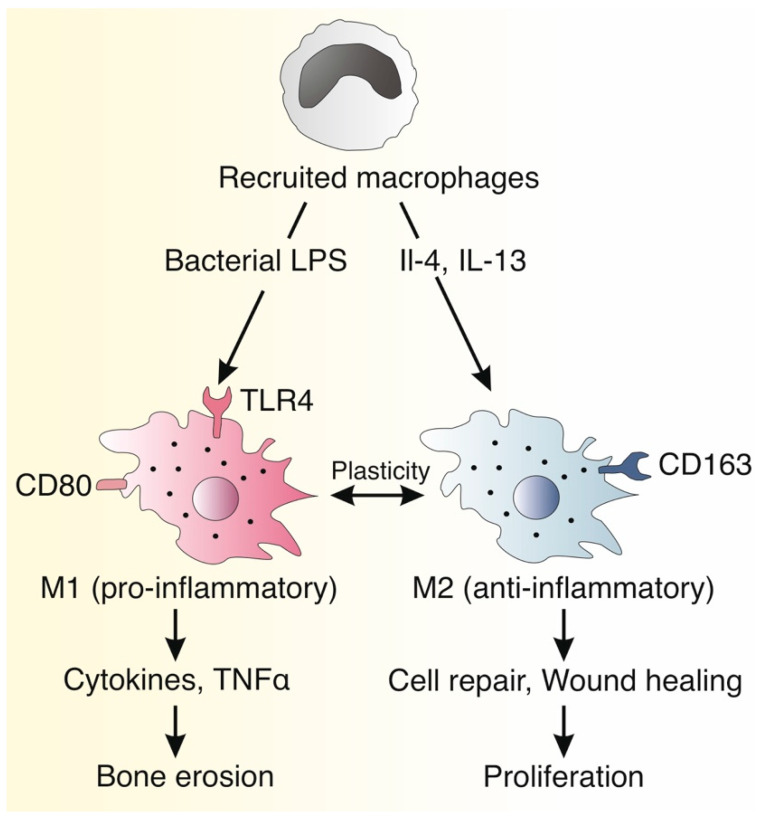
Schematic illustration of the macrophage polarization pathway in middle ear cholesteatoma. Bacterial infection, most commonly with *Pseudomonas aeruginosa*, is commonly found in cholesteatoma since the entrapped keratin is an ideal environment for bacterial biofilms. Recruited macrophages in the middle ear respond to bacterial lipopolysaccharide (LPS), which binds to Toll-like receptor 4, promoting conversion into the proinflammatory M1 macrophage phenotype. In turn, M1 macrophages produce a myriad of inflammatory cytokines, including tumor necrosis factor alpha (TNFα), that induce osteoclastic bone erosion. In contrast, interleukin-4 (IL-4) and interleukin-13 (IL-4) promote conversion to the anti-inflammatory M2 phenotype. M2 macrophages secrete a variety of cytokines and growth factors that contribute to homeostasis, wound healing, and cell repair. These growth factors promote proliferation and cell division. The surface proteins CD80 and CD163 mark the M1 and M2 cells, respectively.

The pathogenesis of bone erosion in cholesteatoma has been previously studied [5,47,48,49,50]. Bone erosion was reported to be caused by matrix metalloproteinase (MMP)-dependent osteoclasts that are stimulated by inflammatory cytokines, most notably tumor necrosis factor alpha (TNFα) [5,47,48,49,50]. In previous studies, the level of TNFα has been correlated with bone erosion in cholesteatoma [51,52]. The production of TNFα is known to be induced by bacterial lipopolysaccharide (LPS), which is present in Gram-negative bacteria such as *Pseudomonas aeruginosa* [53,54,55]. Similarly, LPS was detected at higher levels in cholesteatomas with extensive bone erosion than in those without [56]. Since LPS is also known to bind to Toll-like receptor 4 (TLR4) on the surface of macrophages, promoting their polarization into an M1 phenotype [26,27], we believe our findings link the known aspects of the pathogenesis of bone erosion in cholesteatoma with macrophage polarization in the perimatrix, further establishing acquired cholesteatoma as an inflammatory, immune-mediated disease [57,58,59]. The model is illustrated in Figure 4.

The detection of inflammatory cells, leucocytes, monocytes, and macrophages in human cholesteatoma has been reported in multiple previous studies [5,20,21,22]. However, the M1/M2 macrophage polarization profile in human cholesteatoma has remained uninvestigated until the present study. Macrophage polarization is known to affect disease severity in chronic inflammatory diseases [26,27], such as rheumatoid arthritis [60] and Crohn’s disease [28,29,30]. In addition, pharmacologic manipulation of the M1/M2 polarization has demonstrated therapeutic potential in these diseases [28,60]. In the present study, we revealed a significant association between the macrophage polarization phenotypes and the extent of ossicular erosion. Although M2 macrophages were much more abundant than M1 macrophages in the analyzed cholesteatoma specimens (present study, Figure 2 and Figure 3), the frequency of M1 cells was found to be more relevant to ossicular erosion. Specifically, a higher number of M1 cells (and therefore a higher M1/M2 ratio) was significantly associated with advanced ossicular erosion. Taken together, these results are consistent with the findings of previous studies of other chronic inflammatory diseases [26,27,28,29,30,60].

There are several limitations in the present study. We utilized ossicular erosion as a measure of the clinical aggressiveness of cholesteatomas. However, there are other determinants of disease severity that were not explored in our study, such as cholesteatoma extension and complications. The known staging and classification systems of acquired cholesteatoma (such as the ChOLE [61] or EAONO/JOS [62] classifications) were designed to reflect aspects related to the prognosis from a treatment or surgical standpoint but not biological aggressiveness. For example, the ChOLE staging system describes ossicular status at the end of surgery [61], which is more relevant to the treatment outcome than to the biological disease severity since surgeons may remove an intact ossicle during surgery. In the present study, we decided to employ a simple approach to quantify ossicular erosion based on the number of affected ossicles, which we adapted from the STAMCO classification [36]. While it may be possible to utilize cholesteatoma extension as a marker of clinical aggressiveness, it remains challenging to quantify cholesteatoma extension. The known staging systems emphasize the involvement of difficult surgical areas, such as the sinus tympani, which may lead to a higher risk of residual disease and thus worse treatment outcome, but does not necessarily signify more aggressive biological behavior. Ideally, the true size or volume of cholesteatoma can be used as a marker. However, due to the retrospective nature of our study, we were not able to recapitulate the size of the cholesteatomas in a quantifiable manner.

One further limitation of our study is the oversimplification of macrophage polarization phenotypes by using one M1 and one M2 marker. The current understanding of macrophage biology has revealed that macrophage phenotypes exist as a spectrum or continuum of overlapping gene signatures and thus cannot be encompassed by a single marker or even solely using immunohistochemistry [23,24,25]. Additionally, there exists an inherent selection bias since not all cholesteatomas operated on in our center underwent histopathological tissue analysis, which leads to a bias toward more challenging and/or aggressive cholesteatomas or those with an atypical presentation, as well as the tendency to overlook straightforward cases with mild disease. Although cholesteatoma is reportedly more common in males [63], the frequency of male patients in our cohort was likely even higher than in the general population, probably due to the limited sample size. Increasing the sample size in future studies would probably neutralize or at least reduce this discrepancy. Since the clinical picture of cholesteatoma is not known to vary according to sex, we believe this bias is unlikely to affect the main conclusions of our study. We propose that future studies adopt a prospective design and utilize more elaborate quantification methods to better evaluate the full spectrum of macrophage phenotypes, such as flow cytometric analysis of freshly harvested cholesteatoma cells using multiple antibodies and/or surface markers.

The present study aimed to achieve a better understanding of macrophage regulation in acquired cholesteatoma. The motivation behind our study was two-fold: (1) to provide a biological means to predict and quantify the clinical aggressiveness of cholesteatoma, since this prediction may have implications for disease management, and (2) to explore the possibility of a pharmacological treatment, which still remains an elusive goal. In the authors’ opinion, the most promising therapeutic target appears to be TNFα. The use of TNFα inhibitors, such as infliximab, has been shown to reduce osteoclastic bone resorption in rheumatoid arthritis [64]. The therapeutic effect of infliximab in Crohn’s disease and rheumatoid arthritis has been attributed to a shift from the M1 toward the M2 phenotype, which lowers the M1/M2 ratio [28,60].

Based on the findings of the present study, it is tempting to speculate about a similar potential therapeutic application for infliximab in human acquired cholesteatoma. Interestingly, the only known case report of spontaneous remission of human acquired cholesteatoma in the literature has described complete clinical and radiological disease resolution in a patient under long-term immunosuppression with infliximab [65]. Thus, we hypothesize that a shift from the destructive M1 pathway toward the homeostatic M2 pathway through TNFα inhibition may represent a biological approach to the management of cholesteatoma. However, a potential harmful role of M2 macrophages cannot be excluded in this treatment paradigm and should therefore be investigated experimentally in future studies. A polarization shift toward the M2 phenotype may theoretically lead to uncontrolled cell proliferation and accelerated cholesteatoma growth. Nevertheless, our study lends support to future therapeutic efforts and adds to the current understanding of inflammatory regulation in acquired cholesteatoma. Future studies should explore the potential application of local or systemic TNFα inhibitors, either as the sole therapeutic or in combination with surgery. In particular, intratympanic delivery represents an attractive treatment strategy since it likely results in a higher local concentration in the middle ear and avoids the considerable systemic side effects of TNFα inhibitors. This anti-inflammatory biological treatment paradigm should be tested on human surgical specimens in vitro or using preclinical animal models in vivo as a first step toward clinical translation in the future.

## Figures and Tables

**Table 1:** The average four-frequency pure tone audiometric thresholds at 0.5, 1, 2, and 4 kHz for fewer cholesteatomas with mild (n = 8) and advanced ossicular erosion (n = 20). The *p*-values of the Wilcoxon nonparametric test were used to determine statistical significance.

	Mild Erosion	Advanced Erosion	*p*-Value
Air conduction threshold	43.4 dB (±14.7)	49.6 dB (±19.4)	0.44
Bone conduction threshold	20.7 (±18.5)	21.7 (±16.8)	0.95
Air-bone gap	22.5 (±15.0)	27.8 (±9.5)	0.12

## Data Availability

Supporting data may be obtained from the authors upon request.
